# Sphingolipid Signaling and Complement Activation in Glioblastoma: A Promising Avenue for Therapeutic Intervention

**DOI:** 10.3390/biochem4020007

**Published:** 2024-06-06

**Authors:** Alhaji H. Janneh

**Affiliations:** Hollings Cancer Center, Department of Biochemistry and Molecular Biology, Medical University of South Carolina, Charleston, SC 29425, USA

**Keywords:** glioblastoma, sphingolipids, complement system, tumor microenvironment, therapeutics

## Abstract

Glioblastoma is the most common and aggressive type of malignant brain tumor with a poor prognosis due to the lack of effective treatment options. Therefore, new treatment options are required. Sphingolipids are essential components of the cell membrane, while complement components are integral to innate immunity, and both play a critical role in regulating glioblastoma survival signaling. This review focuses on recent studies investigating the functional roles of sphingolipid metabolism and complement activation signaling in glioblastoma. It also discusses how targeting these two systems together may emerge as a novel therapeutic approach.

## 1. Introduction

**Figure 1.**
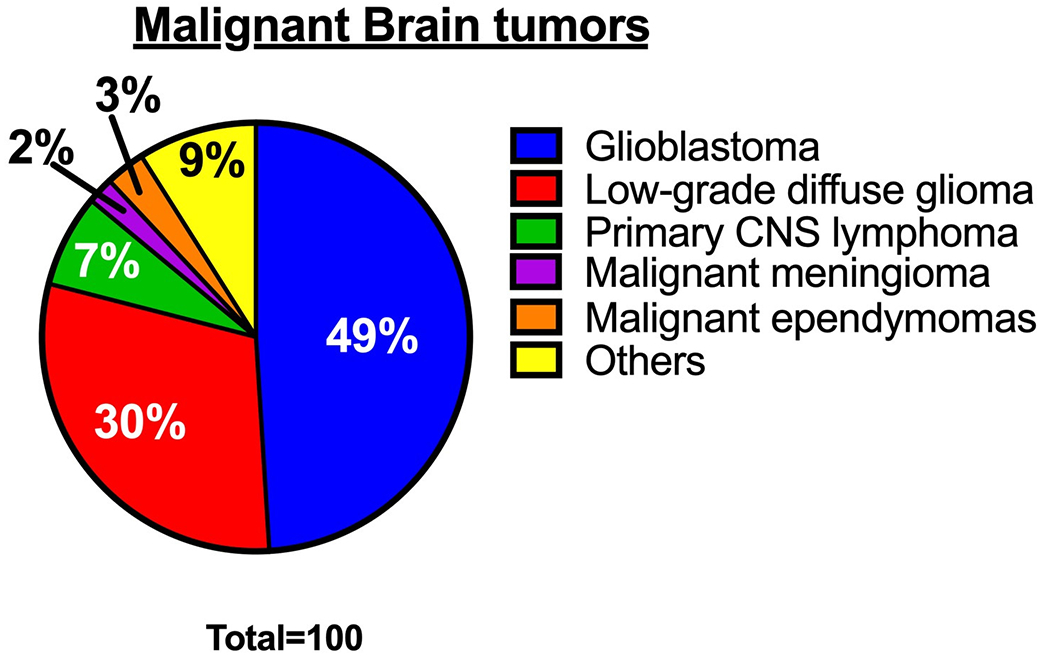
Percentages of malignant brain tumors [1]. The pie chart shows the proportion of various types of malignant brain tumors.

Glioblastoma, also known as glioblastoma multiforme (GBM), is the most common, aggressive, and rapidly growing malignant brain tumor in adults, with a median survival rate of under 24 months [1–3]. Glioblastoma accounts for approximately 49% of all malignant brain tumors (Figure 1) [1] and is mainly composed of abnormal astrocytes. The most common origin of glioblastoma is de novo, arising as a grade 4 tumor [4,5]. An integrative spatial analysis revealed the presence of both disorganized and structured regions in human glioblastoma, with the structured five-layer organization associated with a profusion of hypoxic tumor cells [6]. Interestingly, hypoxic tumor signals can induce hypoxic macrophages in human glioblastoma, stimulating tumor progression [7]. Although glioblastoma can occur at any age, it is more common in older adults and most prevalent in men than women [8]. The World Health Organization (WHO) revised the classification of tumors of the Central Nervous System (CNS) in 2021 [9,10]. According to the revised classification, glioblastoma is now classified as an isocitrate dehydrogenase (IDH) wild-type astrocytoma tumor. This type of tumor has marked nuclear atypia, microvascular proliferation or necrosis, high cellular density, and high mitotic activity, or at least one of the following characteristics: Telomerase reverse transcriptase (TERT) promoter mutation, epidermal growth factor receptor (EGFR) amplification, or concomitant gain of chromosome 7 and loss of chromosome 10 [9,11]. Most cases of glioblastoma are typically found in the frontal lobe, followed by the temporal lobe, parietal lobe, and occipital lobe. Glioblastoma patients typically experience intense headaches, seizures, neurocognitive issues, and focal neurologic deficits [12–14].

The main treatment for glioblastoma involves surgical resection, followed by radiation and chemotherapy [15,16]. The surgical procedure aims to remove as many tumor cells from the brain as possible while preserving the surrounding healthy cells crucial for normal brain functioning [15]. Surgical tumor resection improves patients’ progression-free survival and overall survival [17,18]. For improved survival rates, radiation therapy is given 3 to 6 weeks after surgery, along with oral temozolomide (a DNA-alkylating agent), to target and eliminate any remaining tumor cells that have spread into normal brain tissue [19,20]. During radiation treatment, patients typically receive 60 Gy of radiation delivered in 30 fractions of 2 Gy each over six weeks, targeting the tumor site within a specific margin of infiltrating tumor cells [20,21]. The adjuvant chemotherapy treatment with temozolomide is given to patients four weeks after the completion of radiation therapy for six monthly cycles (5 successive days every 28 days) [22,23]. The O^6^-methylguanine DNA methyltransferase (*MGMT*) gene promoter methylation status is a predictive indicator for patients who will benefit from temozolomide administration [24–26]. Despite the availability of aggressive treatment options, glioblastoma recurrence is inevitable. Unfortunately, patients often undergo a second round of surgery and chemotherapy. The newer therapeutic approaches are less effective against glioblastoma in late-stage clinical trials with no improvement in overall survival [27–29], highlighting the necessity for more effective treatment approaches.

**Figure 2.**
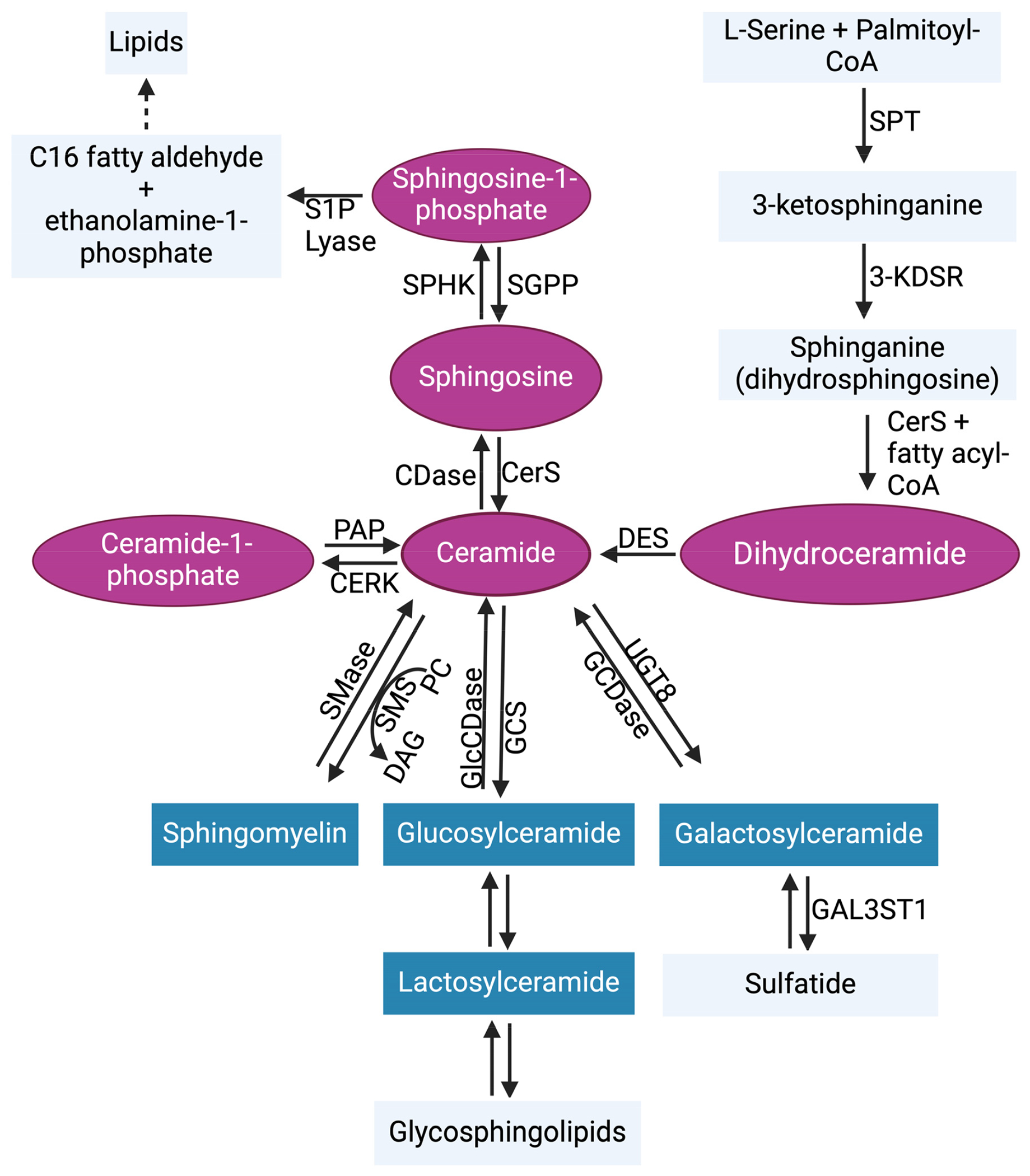
Sphingolipid metabolic pathway. Ceramide is the central hub of sphingolipid biosynthesis. Ceramide can be formed through de novo synthesis, salvage pathway, or through the hydrolysis of complex sphingolipids (blue). In the de novo synthesis pathway, serine palmitoyl transferase (SPT) condenses L-serine + palmitoyl-CoA to generate 3-ketosphinganine (3-keto-dihydrosphingosine). The enzyme 3-ketosphinganine reductase (3-KDSR) then reduces 3-ketosphinganine to generate sphinganine (dihydrosphingosine), which is then converted to dihydroceramide by (dihydro)ceramide synthases 1–6 (CerS1–6). Dihydroceramide desaturase (DES) catalyzes the formation of ceramide by desaturating dihydroceramide. In the salvage pathway, sphingosine can be converted to ceramide by CerS1–6. Ceramide can also be metabolized by ceramidases (CDases) to generate sphingosine, which is phosphorylated by sphingosine kinases 1 and 2 (SPHK1 and SPHK2) to generate sphingosine-1-phosphate (S1P). In reverse, S1P phosphatases (SGPP) can dephosphorylate S1P to reproduce sphingosine. The irreversible actions of S1P lyase can metabolize S1P to yield ethanolamine 1-phosphate and C16 fatty aldehyde, the sphingolipid metabolic pathway exit point. Ceramide kinase (CERK) can phosphorylate ceramide to generate ceramide-1-phosphate, which can also be used to regenerate ceramide by phosphatidate phosphatase (PAP). In the hydrolysis of complex sphingolipids, sphingomyelin can be generated from ceramide by sphingomyelin synthase (SMS), which allows phosphorylcholine transfer to ceramide from phosphatidylcholine (PC) and releasing diacylglycerol (DAG). In return, sphingomyelinases (SMases) cleave sphingomyelin, leading to the release of phosphocholine and ceramide. Additionally, glucosylceramide synthase (GCS) and ceramide galactosyltransferase (UGT8) generate glucosylceramide and galactosylceramide, respectively, from ceramide. This process is also reversible. Abbreviations: GlcCDase, glucosylceramidase; GCDase, galactosylceramidase; GAL3ST1, galactosylceramide sulfotransferase. The pink coloring indicates bioactive sphingolipids, and the blue coloring indicates complex sphingolipids [47].

Sphingolipids, which are abundant in the brain, play a key role in the structure of the plasma membrane and regulate critical biological programs that determine cell fate [30–33]. Sphingolipid metabolism is a complex process that starts from a single common entry point in the de novo metabolic pathway through the enzymatic functions of serine palmitoyl transferase (SPT) [31,34]. At the sphingolipid metabolic exit point, sphingosine-1-phosphate (S1P) lyase 1 irreversibly breaks down S1P to generate C16 fatty aldehyde (hexadecanol) and ethanolamine-1-phosphate products [35,36] (Figure 2). High-performance liquid chromatography (HPLC)-tandem mass spectrometry is the most effective method for monitoring sphingolipid metabolic changes. The liquid chromatography-tandem mass spectrometry (LC-MS/MS) is a highly sensitive and specific method that offers both qualitative and quantitative analysis of sphingolipids in biological samples [37–39]. In addition to LC-MS/MS, other techniques such as the enzymatic-based assays [40,41] or HPLC analysis of fluorescent derivatives [42,43] are less expensive alternatives for sphingolipid measurements but they are not as informative. Several enzymes (Figure 2) are activated to regulate the sphingolipid metabolic pathway by generating important sphingolipids like ceramides, which mediate cancer cell death, and S1P, which promotes tumor survival. The significant roles of sphingolipids in cancer progression have been well reviewed previously [30,44–46]. Bioactive sphingolipids such as S1P facilitate communication with other regulatory programs. These programs include the complement system in innate immunity, and they collaborate to promote cancer cell signaling.

**Figure 3.**
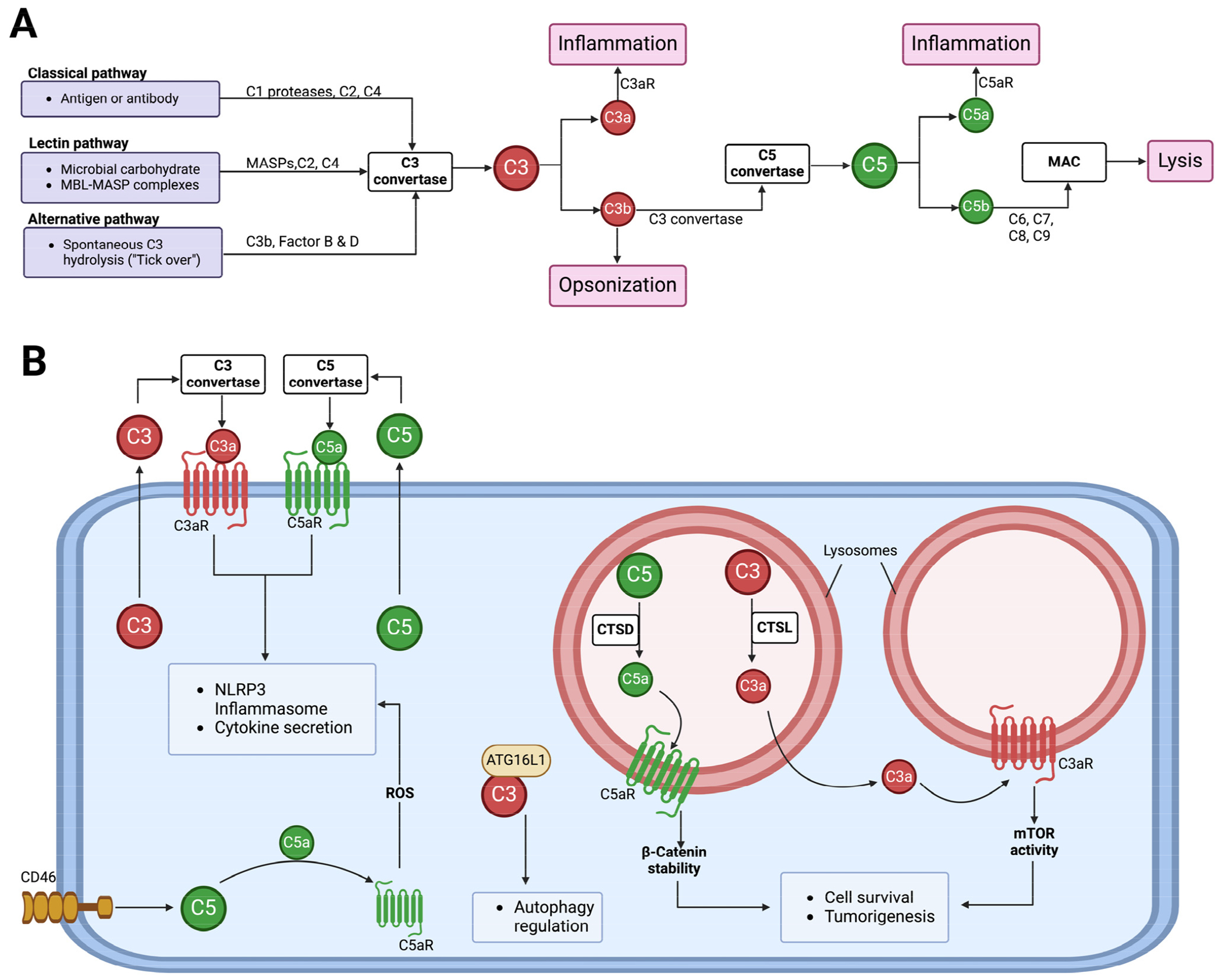
The activation of the complement system and its functions. (**A**) Complement from the liver into circulation can be activated via three canonical pathways, namely classical, lectin, and alternative. All three pathways lead to the formation of C3 and C5 convertases. C3 convertase cleaves C3 into biologically active C3a and C3b, and C5 convertase cleaves C5 into biologically active C5a and C5b. The C5b fragments initiate the formation of the membrane attack complex (MAC), which functions to cause osmotic lysis. Other complement component functions include C3b acting as an opsonin, while C3a and C5a anaphylatoxins induce inflammation via C3aR and C5aR, respectively. (**B**) Cells can secrete C3 and C5 components to the extracellular space, where they get activated by C3 and C5 convertases, respectively. The activated fragments, C3a and C5a, can signal via their respective receptors to induce NLRP3 inflammasome formation and cytokine release. Stimulation of the CD46 receptor can activate intracellular C5, allowing C5a–C5aR1 signaling to induce the production of reactive oxygen species (ROS) rnd NLRP3 assembly [66]. Intracellular C3 ban regulate autophagy by binding to autophagy-related protein 16-1 (ATG16L1) [67]. Intracellular complement activation can also occur in subcellular compartments. In lysosomes, cathepsin L (CTSL) and cathepsin (CTSD) can activate C3 and C5, respectively, to promote cell survival and tumorigenesis [55,57].

The complement system is a key functional component of the innate immune system and is also important in adaptive immunity [48]. A group of proteins, both plasma and membrane-bound, come together to form the complement components that detect and combat pathogens [48–51]. Complement activation occurs via canonical/conventional pathways and non-canonical mechanisms [52] (Figure 3). The canonical activation pathways involve the assembly of protein complexes and the initiation of enzymatic cascades that lead to cleavage reactions via the classical, lectin, or alternative pathways [53]. The non-canonical mechanisms involve the activation of complement components in intracellular compartments of both immune and non-immune cells, which has shifted our understanding of this ancient component of the immune system [54]. Proteases like cathepsin L (CTSL) and cathepsin D (CTSD) are capable of activating intracellular complement components to generate biologically active fragments like C3a/C3b and C5a/C5b [55–57]. Studies in the 1990s revealed that pro-CTSL secreted by human melanoma cells cleaves exogenous C3, promoting tumor growth and metastasis [58,59]. However, there was no evidence of C3 being processed into biologically active C3a and C3b in these studies. Recent studies have now shown that complement-activating products can regulate the tumor microenvironment to promote tumor growth/survival [60–62]. The role of complement-activating products in tumor regulation is context-dependent, as thoroughly reviewed in 2019 [61]. A complete understanding of the complement activation process will likely continue to lead to the discovery of new connecting links between the activating components of the complement system and other effector systems to regulate cellular functions [63–65].

This review explores the critical roles of sphingolipids and complement-activating products in regulating glioblastoma and highlights the potential of targeting both pathways together as a more effective therapeutic approach.

## 2. Sphingolipid Metabolism in Glioblastoma

The sphingolipid metabolic pathway is altered during the progression of glioblastoma tumors due to the enrichment of lipids in the brain [68–70]. Sphingolipids, such as ceramides and sphingosine-1-phosphate (S1P), play a crucial role in the regulation of glioblastoma growth and therapeutic resistance.

### 2.1. Ceramides

Ceramide is the central hub molecule in the sphingolipid metabolic pathway. It can be synthesized through the enzymatic functions of Ceramide Synthases 1–6 (CerS1–6) [71,72]. CerS1–6 and the consequent production of ceramides have been linked to apoptosis [73–75]. Ceramides can also be generated through the hydrolysis of sphingomyelin, a process that is catalyzed by Sphingomyelinases (SMases) [76].

**Figure 4.**
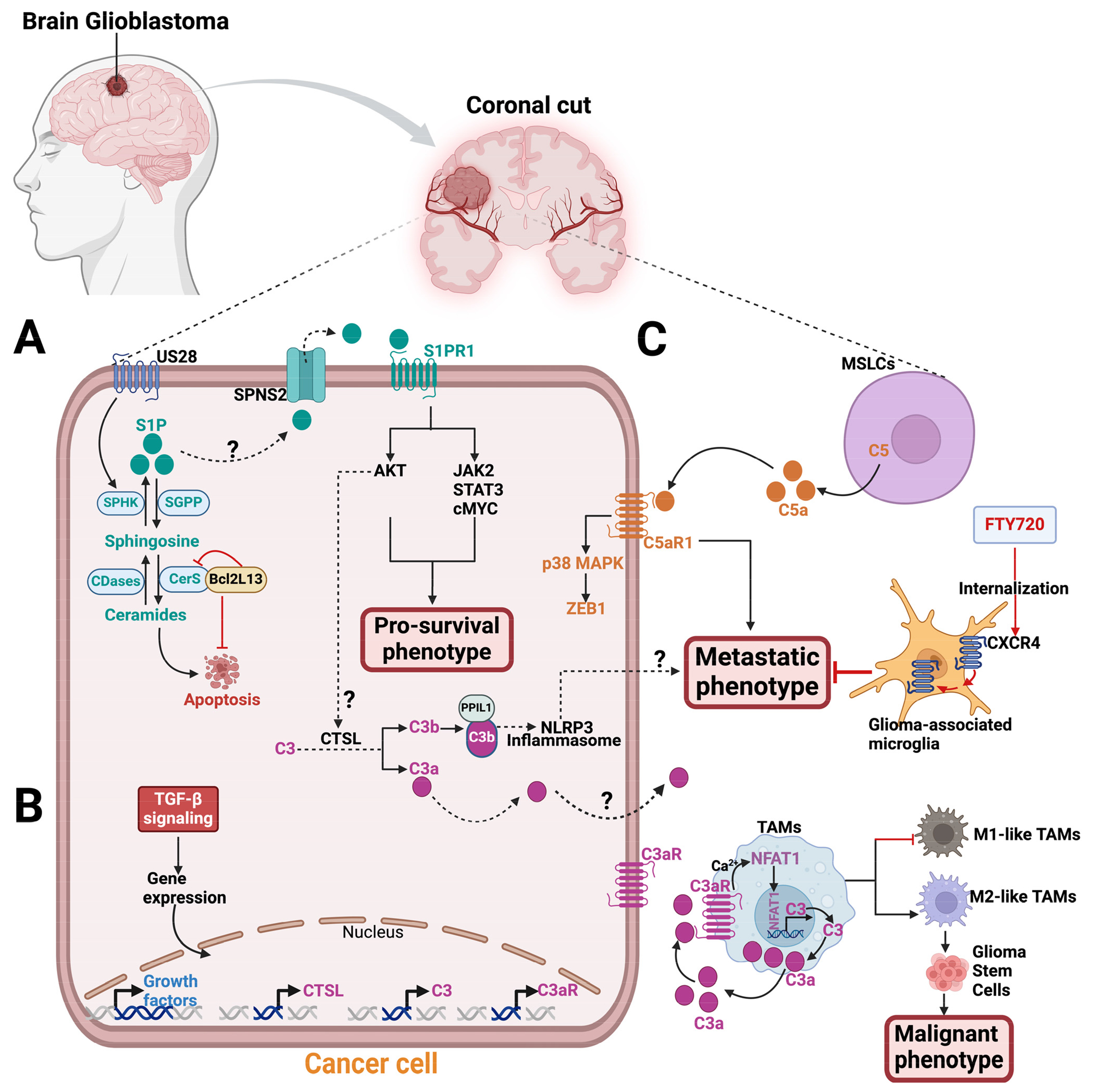
Sphingolipids and complement components signaling in glioblastoma. A zoomed coronal plane view of a glioblastoma tumor and its microenvironment. (**A**) In glioblastoma tumor, the US28 receptor stimulates SPHK1 in the sphingolipid metabolic pathway to release S1P, which exits the cell through an S1P transporter, like SPNS2, to engage with S1PR1. S1P/S1PR1 signaling activates AKT, JAK2, STAT3, and cMYC pathways to promote glioblastoma pro-survival phenotype. A study has shown that the S1P-S1PR1 signaling pathway activates AKT, which in turn triggers intracellular C3 cleavage into biologically active C3a and C3b via CTSL protease [56]. This activation enables PPIL1-C3b binding that induces NLRP3 inflammasome. The result of this process is the development of metastatic phenotype in melanoma, breast, and head and neck cancers. This mechanism may also be applicable to glioblastoma. The mitochondria-associated protein, Bcl2L13, is upregulated in glioblastoma and binds CerS2 and CerS6, inhibiting apoptosis by blocking ceramide synthesis. (**B**) Exposing glioblastoma to TGF-β increases mRNA expression levels for C3aR, C3, CTSL, and growth factors. (**C**) In the tumor microenvironment, mesenchymal stem-like cells (MSLCs) secrete the C5a anaphylatoxin, which binds to the C5aR expressed on glioblastoma tumors. C5a–C5aR1 signaling stimulates a metastatic phenotype on glioblastoma by increasing ZEB1 expression via the p38 MAPK pathway. FTY720 treatment internalizes CXCR4 on glioma-associated microglia to inhibit the metastatic phenotype of glioblastoma cells. In tumor-associated macrophages (TAMs), the nuclear factor of activated T cells-1 (NFAT1) stimulates C3 transcriptional activity and increases C3a secretion, which binds C3aR in an autocrine manner. In a positive feedback loop, C3a-C3aR signaling activates the Ca^2+^-NFAT1 pathway, which induces M2-like TAMs and promotes glioma stem cells (GSCs) malignant phenotype. Abbreviations: SPHK1, sphingosine Kinase 1; S1P, sphingosine-1-phosphate; SPNS2, spinster homologue 2, S1PR1, sphingosine-1-phosphate receptor 1; CTSL, cathepsin L; PPIL1, Peptidylprolyl Isomerase Like 1, NLRP3, NLR Family Pyrin Domain Containing 3; ZEB1, Zinc finger E-box binding homeobox 1.

Radiation and chemotherapy drugs can induce cell death by apoptosis through ceramide accumulation from sphingomyelin conversion catalyzed by SMases [77–79]. An analysis of human tumor samples using quantitative ceramide measurement revealed that low ceramide levels in glioblastoma patients were linked to malignant tumor progression and poor patient survival. This suggests that decreased ceramide levels may confer a growth advantage to glioblastoma tumors by providing apoptotic resistance [80]. Interestingly, in a glioblastoma xenograft model, the mitochondria-associated Bcl2-like 13 (Bcl2L13) protein, a member of the Bcl-2 family, was found to promote glioblastoma growth by inhibiting apoptosis through decreasing the ceramide levels in response to chemotherapy treatment [81]. Bcl2L13 was shown to be overexpressed in glioblastoma tumors and binds CerS2 and CerS6, which inhibits the de novo synthesis of ceramides [81] (Figure 4). IL-24, a cytokine that triggers apoptosis, was reported to stabilize CerS6, inducing ceramide synthesis, reactive oxygen species (ROS) production, and Ca^2+^ elevation to promote human glioblastoma cell death in response to endoplasmic reticulum (ER) stress [82,83]. ER stress was also activated by CerS1 overexpression or C18-ceramide accumulation, which induces glioblastoma cell death via lethal autophagy in A172 and U251 human glioblastoma cell lines [84]. Although ceramide accumulation in glioblastoma can induce apoptosis, tumor cells are able to evade this process by converting ceramides to S1P, which is a pro-survival signaling molecule.

### 2.2. Sphingosine-1-Phosphate (S1P) Signaling

S1P is a bioactive sphingolipid that is generated intracellularly by sphingosine kinases 1 and 2 (SPHK1 and SPHK2). SPHK1-generated S1P in the cytoplasm can exit the cell via specific S1P transporters to engage with the five known S1P receptors (S1PR1-5), leading to an “inside-out” signaling process known to occur in tumors [44,85–87].

Sphingolipid quantification by liquid chromatography with tandem mass spectrometry revealed a sphingolipid metabolic shift favoring S1P production over ceramide in human glioblastoma tissue samples [88]. S1P levels were nine times higher, while the most abundant ceramide in the brain (C18-ceramide) was five times lower in human glioblastoma tissues compared to normal human gray matter. The elevated S1P levels in the tumors were consistent with increased SPHK1 expression [88]. Unlike S1PR4, S1PR1,2,3, and 5 have been reported to be expressed in glioblastoma cells and regulate S1P signaling with context-dependent effects on tumor progression [89–94]. As previously reviewed, S1PR1 has been reported as a promising therapeutic target for cancer treatment [95,96]. The human cytomegalovirus-encoded G protein-coupled receptor (GPCR), US28, was reported to promote U251 glioblastoma malignancy by stimulating SPHK1 function to release more S1P, which signals via S1PR1 using in vitro assays (Figure 4). The SPHKI/S1P/S1PR1 signaling activates AKT, JAK2/STAT3, and cMYC and enhances the levels of the cancerous inhibitor of protein phosphatase 2A (CIP2A) downstream to promote the pro-survival phenotype in glioblastoma cells [97]. Using xenogeneic glioma mouse models and in vitro assays, Arseni et al. showed that SPHK1/S1P/S1PR signaling axis consistently stimulates the enhanced recruitment of tumor-associated macrophages (TAMs), triggering pro-tumorigenic phenotype in glioblastoma cells [98]. Thus, a putative S1PR1 modulator, ACT-209905, inhibits the growth and migration of human and mouse glioblastoma cell lines in vitro [99]. However, analysis of fresh human glioblastoma tissues from 117 patients who underwent surgical resections revealed that S1PR1 expression was associated with extended patient survival, while high S1PR2 expression was linked to a poorer survival outcome [89]. Nonetheless, in the U-118 MG and U-373 MG human glioblastoma cell lines, both S1PR1 and S1PR3 concurrent expression promote the motility and invasiveness of glioblastoma cells through overlapping but distinct mechanisms in vitro [100,101]. The authors suggest that S1PR1 alone is insufficient for a maximum S1P-induced response in the glioblastoma cell lines [100,101].

## 3. Complement Signaling in Glioblastoma

The tumor microenvironment’s complex and heterogeneous composition comprises the activating components of the complement system, which regulates the growth of several tumor types, including glioblastoma. The complement proteins, which can be locally synthesized in the brain [102], can be hijacked by glioblastoma cells to facilitate tumor growth [103–105]. The tumor microenvironment of glioblastoma includes astrocytes, oligodendrocytes, neurons, immune cells (macrophages, neutrophils, monocytes, T cells, etc.), brain vascular system, extracellular matrix layers, glioma, and glioma stem cells [106]. The complement components, expressed in cells within the tumor microenvironment, as well as chemicals (oxygen, pH, etc.) and other components that aid cell-to-cell communication, can facilitate the growth of glioblastoma.

Interestingly, immunohistochemistry analysis revealed that human glioblastoma tissues exhibit local complement activation, as evidenced by the deposition of complement products such as C1q, C3c, C4d, and the C5b-9 terminal complex [107–109]. Abnormal levels of components in the classical pathway of the complement system were detected via either electro-immunoassay, enzyme-linked immunosorbent assay (ELISA), or nephelometry techniques in the peripheral blood of patients with *IDH*-wild-type glioblastoma [110]. Single-cell RNA sequencing from five primary glioblastoma cells showed intra-tumoral heterogeneity and expressed complement pathway genes, including C3 [111]. Additionally, human glioblastoma tumors and their tumor-associated macrophages (TAMs) showed robust levels of C3a and C3aR, respectively, to promote tumor survival through the alternative pathway [112]. Exposing human glioblastoma cells to Transforming Growth Factor-β (TGF-β) for 24 h resulted in increased mRNA levels of C3, C3aR, CTSL, and other growth factors [112] (Figure 4). The elevated CTSL mRNA level may suggest intracellular complement activation in glioblastoma tumors. Also, in mice, activation of the nuclear factor of activated T cells-1 (NFAT1) increases the transcription activity of C3, leading to the secretion of C3a. C3a then binds to its receptor, C3aR, resulting in a positive feedback loop that promotes M2-like TAMs, which in turn promote the malignant phenotype of glioma stem cells [113]. The growth of glioma was hindered by the blockade of the NFAT1-C3a-C3aR axis using a C3aR inhibitor [113].

Remodeled mesenchymal stem cells (MSCs), known as mesenchymal stemlike cells (MSLCs), found in the stromal components of glioblastoma and many other tumor types, were reported to secrete C5a anaphylatoxin [103,114,115]. This secretion promotes the invasion/infiltration of glioblastoma cells into the parenchymal brain tissue of mice [103]. Mechanistically, MSLCs in mice glioblastoma microenvironment secrete C5a, which engages with C5aR1 expressed in glioblastoma cells in a paracrine manner. The activation of the C5a–C5aR1 signaling axis on the glioblastoma cells increases the expression of ZEB1, a regulator of epithelial–mesenchymal transition (EMT), via the p38 MAPK pathway, promoting invasion/infiltration of glioblastoma cells into parenchymal brain tissue [103] (Figure 4). Targeting C5a in malignant MSLCs isolated from glioblastoma patients can potentially lead to improved survival outcomes for these patients.

Glioblastoma leptomeningeal spread/metastasis, a severe complication of glioblastoma, occurs when tumors infiltrate the cerebrospinal fluid (CSF) and leptomeninges from the brain parenchyma [116–118]. Interestingly, the upregulation of C3 was shown to promote leptomeningeal metastasis [119]. The study revealed that tumor cells present in the CSF produce high levels of biologically active C3a. This C3a binds to the C3aR on the choroid plexus, which weakens or impairs the choroidal blood–CSF barrier. As a result, growth factors such as amphiregulin can enter the CSF from the circulation and promote tumor growth and metastasis [119]. Blocking C3aR prevented leptomeningeal metastasis in mice, making the C3a–C3aR signaling axis a target for a potential therapeutic strategy to prevent leptomeningeal metastasis [119].

## 4. Crosstalk between Sphingolipid Metabolism and Complement Signaling

A few studies have suggested a context-dependent interaction between sphingolipid metabolism and the complement system. For example, in mice models of graft versus leukemia, complement anaphylatoxin receptors (C3aR and C5aR) signaling in dendritic cells regulates ceramide-dependent lethal mitophagy. This process is essential for the development of graft-versus-host disease (GVHD) after hematopoietic cell transplantation (HCT). If the activation of C3aR–C5aR signaling in the recipient dendritic cells is blocked, it increases ceramide generation and trafficking, which enhances lethal mitophagy. This, in turn, alleviates GVHD outcomes while maintaining the effect of graft versus leukemia [120]. Activation fragments of the complement cascade, such as C3a, desArgC3a, C5a, desArgC5a, and C5b-C9 MAC, regulate the movement of hematopoietic stem progenitor cells by modulating their migration functions. This increases the levels of S1P and ceramide-1-phosphate (C1P) chemoattractants [121–124]. Moreover, C5a–C5aR signaling activates SphK1 expression through the p38 MAPK pathway in mice experiencing acute liver failure [125]. Blocking C5a–C5aR signaling lowers SphK1 and serum S1P, preventing acute liver dysfunction in mice [125]. Consistently, C5a was shown to activate SphK1–S1P signaling in experimental lung inflammatory injury, and the genetic deletion of SphK1 in mice repressed C5aR2 (C5L2) expression on neutrophils [126]. Exogenous S1P can restore C5aR2 expression in mice lacking SphK1, which can help reduce lung inflammation and injury [126].

Additionally, a protein called ceramide transporter protein (CERT) plays a crucial role in the maintenance of normal sphingolipid levels in cells by facilitating the movement of ceramide from the endoplasmic reticulum to the trans-Golgi apparatus. Recent reports have revealed that CERT also has the ability to bind to C1q, a protein that initiates the classical pathway-dependent complement activation [127,128]. This study suggests a possible role for CERT and C1q in the clearance of apoptotic cells [128]. In Gaucher disease, a lysosomal storage disorder linked to malignant cancers, the accumulation of glucosylceramide and inflammatory response is regulated by the classical pathway-dependent complement activation through C5a–C5aR1 signaling [129,130].

## 5. Targeting Sphingolipids and the Complement System in Glioblastoma

Given that sphingolipids and complement components play crucial roles in glioblastoma biology and that glioblastoma patients experience the inevitable tumor recurrence after standard-of-care treatment, targeting the sphingolipid metabolic pathway and the complement system may be a novel combination treatment strategy. Interestingly, the crosstalk between sphingolipid signaling and intracellular complement activation in promoting solid tumor survival has been established recently in mouse melanoma, breast, and human head and neck cancer cell lines [56]. Mechanistically, oncogenic S1P–S1PR1 signaling via the AKT pathway activates intracellular C3 mediated by CTSL, which cleaves C3 into C3a and C3b. An activating product of C3b forms a complex with PPIL1 (Peptidylprolyl Isomerase Like 1), which induces NLRP3 inflammasome formation, as evidenced by caspase-1-dependent IL-1β activation, to promote tumor metastasis [56]. Additionally, the C3a secreted from the tumors could also engage with C3aR1 in the tumor microenvironment to promote NLRP3 inflammation-induced metastasis [48] (Figure 4). Inhibitors for both the complement and sphingolipid pathways are available in either the market or clinical trials, and they could be used as a combination therapy to treat glioblastoma [48].

Fingolimod (FTY720), a structural analog of sphingosine, is an FDA-approved drug for multiple sclerosis that acts as a functional antagonist for S1PR1 in its phosphorylated form, p-FTY720 [131–133]. The SPHK2 enzyme phosphorylates FTY720, which leads to the degradation of lymphocyte S1PRs by internalization, thereby preventing S1P–S1PR signaling, which inhibits normal lymphocyte egression from lymphoid tissues [44,134]. Intriguingly, in glioma-bearing rats, FTY720 can internalize CXCR4 on glioma-associated microglia and macrophages in the tumor microenvironment to suppress the migration/invasion of C6 glioma cells by preventing MAPK-mediated IL-6 release [135] (Figure 4). FTY720 can also induce apoptosis and anti-proliferative effects in human glioblastoma cells to prevent tumor progression [136–139]. Combining temozolomide with FTY720 demonstrated an increased apoptotic effect on glioblastoma, resulting in improved survival rates in glioblastoma mouse models [140].

In addition to its S1P signaling inhibiting function, FTY720’s ability to cross the blood–brain barrier makes it promising for treating glioblastoma. Thus, combining FTY720 with C3 pathway inhibitors could be a promising treatment strategy. Complement inhibitors in clinical trials that block the central complement activation pathways include APL-9 [141] (NCT04402060) and AMY-101 [142,143] (NCT03694444 and NCT04395456). Inhibitors that target downstream C5 signaling include ravulizumab (NCT05644561), eculizumab (NCT00112983), vilobelimab (NCT04812535), and avdoralimab (NCT04563923 and NCT04333914). Blocking the complement pathway at an early stage can be achieved by using C1 esterase inhibitors such as Ruconest to block the classical pathway. The lectin activation pathway can also be targeted using a MASP2-specific antibody called narsoplimab (NCT02682407). Combining these complement inhibitors with other sphingolipid drugs or inhibitors is possible. For instance, combining ceramide nanoliposomes (CNLs), which selectively induce cancer cell death via ceramide accumulation [44,144,145], with a complement inhibitor may provide a promising treatment strategy for glioblastoma. Also, inhibiting SPHK2 with opaganib/ABC294640 (NCT04207255, NCT02757326, NCT03377179, and NCT03414489) or SPHK1 with safingol (NCT00084812) in combination with a complement inhibitor could prove to be a novel treatment strategy. In fact, there have been several reports highlighting the role of sphingosine kinases in glioblastoma [146–153], suggesting a rationale for targeting these enzymes.

However, the complement inhibitors discussed above only target the canonical complement activation pathway, which has surprisingly been less successful than expected. The lack of success with the extracellular complement drug targets suggests that intracellular or non-canonical complement activation inputs should not be overlooked. Therefore, it is crucial for new complement pathway inhibitors to effectively target both the canonical and non-canonical activation pathways. CTSL, which cleaves intracellular C3 into biologically active C3a and C3b [55,56], has been reported to contribute to glioblastoma malignancy [154–157], which supports the role of proteases in mediating glioblastoma invasiveness [158]. Thus, inhibition of CTSL activity reduced glioblastoma cell survival and increased cell death via apoptosis in vitro using human glioma cell lines [154]. Therefore, targeting CTSL can prevent intracellular C3 cleavage, which can enhance complement and sphingolipid combination therapy effectiveness.

A combination therapy for glioblastoma should also target the NLRP3 inflammasome pathway, which induces tumor metastasis in response to S1P/S1PR1 and C3 complement signaling [56]. NLRP3 was found to promote cell survival and invasion in human glioma cell lines through IL-1β and NF-κB p65 [159]. Therefore, suppressing NLRP3 expression and inflammation can inhibit glioblastoma’s potential for malignancy [159–162].

## 6. Conclusions and Future Directions

Despite significant advances in research technologies, glioblastoma still stands as the most aggressive form of brain cancer with a dismal outlook for survival, primarily due to its inherent intra-tumor heterogeneity. The complex interaction between glioblastoma tumors and their microenvironment, which includes immune and non-immune cells, increases intra-tumor heterogeneity. To fully understand the most effective strategy to treat glioblastoma, we must investigate its interaction with the tumor microenvironment and the biological systems regulating this interaction. Targeting sphingolipid molecules as a potential therapy for glioblastoma is beneficial. This is because they are specific molecular targets capable of restoring anti-tumor immune functions. Sphingolipids are important regulators of various cellular processes. Therefore, manipulating them in combination with other key biological processes, like the complement system, could be a more effective approach for a new targeted treatment strategy. Additionally, there are numerous sphingolipid-based anti-cancer drugs currently in late-stage clinical trials that could be easily utilized in a new targeted combination therapy for treating glioblastoma.

The complement system and the sphingolipid metabolic pathway signaling can promote tumor survival by activating pro-tumorigenic immune and non-immune cells in the tumor microenvironment. As discussed above, sphingolipids and the activating components of the complement system have been shown to have pro-tumorigenic functions in glioblastoma tumors. Therefore, understanding the crosstalk between sphingolipids and the complement system may emerge as an effective therapeutic approach for treating glioblastoma. However, monitoring the inflammatory effect of targeting both sphingolipids and complements in potential combination therapy is crucial, as they can both mediate inflammatory signals. It has been reported that NLRP3 inflammasomes are regulated downstream of complement signaling [56,66,163]. Thus, targeting NLRP3 in a sphingolipid-complement formulated therapy should be considered.

## Data Availability

Data is contained within the article.
